# Thiol-based chemical probes exhibit antiviral activity against SARS-CoV-2 via allosteric disulfide disruption in the spike glycoprotein

**DOI:** 10.1073/pnas.2120419119

**Published:** 2022-01-24

**Authors:** Yunlong Shi, Ari Zeida, Caitlin E. Edwards, Michael L. Mallory, Santiago Sastre, Matías R. Machado, Raymond J. Pickles, Ling Fu, Keke Liu, Jing Yang, Ralph S. Baric, Richard C. Boucher, Rafael Radi, Kate S. Carroll

**Affiliations:** ^a^Department of Chemistry, Scripps Research, Jupiter, FL 33458;; ^b^Departamento de Bioquímica, Facultad de Medicina and Centro de Investigaciones Biomédicas, Universidad de la República, Montevideo 11800, Uruguay;; ^c^Department of Epidemiology, University of North Carolina at Chapel Hill, Chapel Hill, NC 27599;; ^d^Protein Engineering Unit, Institut Pasteur de Montevideo, Montevideo 11400, Uruguay;; ^e^Marsico Lung Institute, University of North Carolina at Chapel Hill, Chapel Hill, NC 27599;; ^f^Department of Microbiology and Immunology, University of North Carolina at Chapel Hill, Chapel Hill, NC 27599;; ^g^State Key Laboratory of Proteomics, Beijing Proteome Research Center, National Center for Protein Sciences, Beijing Institute of Lifeomics, Beijing 102206, China

**Keywords:** redox biology, SARS-CoV-2, spike glycoprotein, disulfide bonds, thiol-based chemical probes

## Abstract

The development of small-molecules targeting different components of SARS-CoV-2 is a key strategy to complement antibody-based treatments and vaccination campaigns in managing the COVID-19 pandemic. Here, we show that two thiol-based chemical probes that act as reducing agents, P2119 and P2165, inhibit infection by human coronaviruses, including SARS-CoV-2, and decrease the binding of spike glycoprotein to its receptor, the angiotensin-converting enzyme 2 (ACE2). Proteomics and reactive cysteine profiling link the antiviral activity to the reduction of key disulfides, specifically by disruption of the Cys379–Cys432 and Cys391–Cys525 pairs distal to the receptor binding motif in the receptor binding domain (RBD) of the spike glycoprotein. Computational analyses provide insight into conformation changes that occur when these disulfides break or form, consistent with an allosteric role, and indicate that P2119/P2165 target a conserved hydrophobic binding pocket in the RBD with the benzyl thiol-reducing moiety pointed directly toward Cys432. These collective findings establish the vulnerability of human coronaviruses to thiol-based chemical probes and lay the groundwork for developing compounds of this class, as a strategy to inhibit the SARS-CoV-2 infection by shifting the spike glycoprotein redox scaffold.

## Significance

Some coronaviruses utilize angiotensin-converting enzyme 2 (ACE2) for entry into host cells. Although reducing agents, such as *N*-acetylcysteine, disrupt viral binding to ACE2 in general, these compounds are cytotoxic, have low potency, and because of their membrane permeability, have undefined mechanism of action. With qualitative chemoproteomic mapping to delineate cysteine thiol/disulfide reactivity in native spike and recombinant receptor binding domain (RBD), we report nontoxic, cell-impermeable thiol-based chemical probes that significantly decrease the ACE2 binding and infectivity of SARS-CoV-2. We map the reactive cysteines and show the dynamic consequences of breaking allosteric disulfide bonds in the RBD. Altogether, our work underscores a clear redox-based mechanism of antiviral activity in which reducing compounds disrupt key RBD disulfides specifically in extracellular spaces.

Control of pandemics requires rapid and sensitive testing, widely available vaccines, and effective therapeutic agents for the infected. For the current SARS-CoV-2 pandemic, impressive progress in the development and testing of vaccines has been achieved. Although early development of effective SARS-CoV-2 antivirals inhibiting viral polymerase or main protease has reported limited clinical efficacy (1, 2), very impressive results have recently been communicated (3–5). Many other antiviral strategies have focused on the intricate SARS-CoV-2 cellular entry processes that include interactions of the spike glycoprotein’s receptor-binding domain (RBD) with the angiotensin-converting enzyme 2 (ACE2), cell surface proteases, and complex fusion events (6, 7). The development of antiviral agents that target the receptor binding and entry processes requires precise knowledge of the dynamic structures of the viral elements that mediate the binding and entry of virus into target cells (8, 9).

Disulfide bond formation is central to the dynamic structure of many viral receptor binding and entry/fusion proteins (10). The role of disulfide bonds in cognate receptor binding proteins (i.e., spike proteins) has been widely studied in coronaviruses, including mouse hepatitis virus, SARS-CoV, and SARS-CoV-2 (11–13). Viral disulfides are initially formed in the endoplasmic reticulum. These bonds, important for both virus binding and fusion, are further stabilized by the oxidizing extracellular milieu (10, 13). The SARS-CoV-2 RBD contains four disulfide pairs: one disulfide Cys480–Cys488 situated at the receptor binding motif (RBM), and three others Cys336–Cys361, Cys379–Cys432, Cys391–Cys525 to stabilize the β-sheet structure (8). The position of these disulfides in RBD crystal structures has led to speculation that reduction of these bonds may have untapped utility for chemical probe targeting (14–18). However, the precise function of these disulfide pairs cannot be read from structure alone, and cysteine reactivity mapping has not been performed to investigate this hypothesis.

**Fig. 1.**
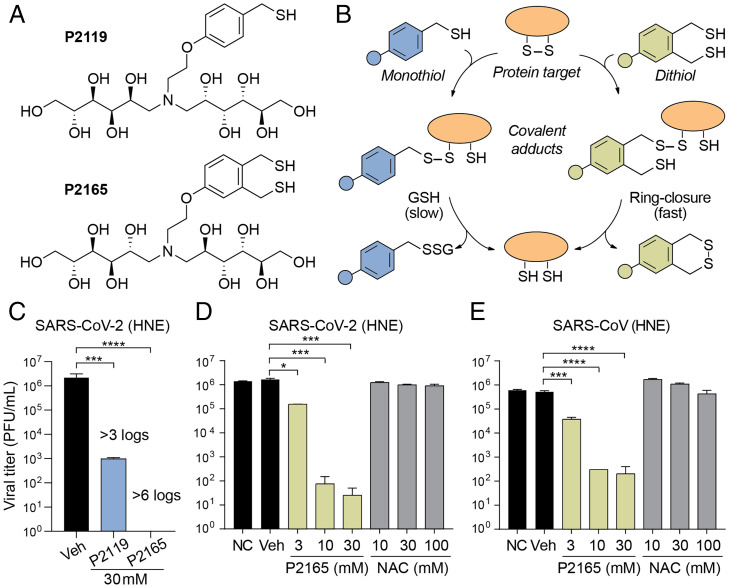
Thiol-based reducing agents have potent virucidal activity against human coronaviruses. (*A*) Chemical structures of P2119 and P2165 reducing agents. (*B*) Mechanisms of disulfide reduction by thiol-based reducing agents. Adducts formed with P2119 require a second thiol equivalent (e.g., from GSH or drug) to resolve the mixed disulfide, whereas the adduct formed with P2165 rapidly undergo intramolecular thiol-disulfide exchange. (*C*–*E*) Comparison of 72-h titers between nontreated control (NC), PBS vehicle (Veh), or reducing agents-treated SARS-CoV or SARS-CoV-2 infected primary HNE cultures at a multiplicity of infection (MOI) of 0.1. Triplicated titers of the virus in cultures from the same donor were analyzed by ANOVA with Dunnett’s test. *P < 0.05; ***P < 0.001; ****P < 0.0001.

The preclinical thiol-based reducing agents P2119 and P2165 (Fig. 1*A*) are useful tools to test the potential utility of this class of compounds in pulmonary diseases (19–21). These compounds were selected from a library of glycosylated thiols designed to both exhibit higher intrinsic reducing activity than currently available agents and restrict their molecular activity to the extracellular space (i.e., not membrane permeant), to provide durable activity on airway surfaces and protect against cellular toxicity. Accordingly, P2119 and P2165 exhibit a greater therapeutic index compared to clinically available therapeutics, including *N*-acetylcysteine (NAC) (22, 23), which has a low intrinsic activity and is taken up by the cell and metabolized to hydrogen sulfide (H_2_S) (24), a second messenger that initiates pleiotropic changes in myriad targets (25). P2119 and P2165 also afford an opportunity to compare the intrinsic potency of a monothiol versus a dithiol compound, and their associated mechanisms of action as antiviral agents (Fig. 1*B*).

Here, we show that thiol-based chemical probes P2119 and P2165, which function as reducing agents, inhibit infection by human coronaviruses, including SARS-CoV-2, and decrease the binding of spike glycoprotein to its receptor, ACE2. The antiviral activity of these thiol-based reducing agents is linked to the reduction of disulfides in the RBD of spike glycoprotein. Proteomics and reactive cysteine mapping show that the Cys480–Cys488 pair, located in the loop region of the ACE2 binding surface, is not susceptible to alkylation during live cell infection, establishing the stability of this disulfide in a native setting. In contrast, Cys432 and Cys525, which form disulfides with Cys379 and Cys391, respectively, were identified as hyperreactive cysteines that form semistable disulfides. Molecular docking analysis provides insight into the targeting of Cys432 by both reducing agents. Molecular dynamics (MD) simulations predict that reduction of these three disulfides controls ACE2 binding by triggering conformational changes in the RBD. The latter finding suggests that Cys379–Cys432 and Cys391–Cys525 bonds are allosteric, a unique and rare category of disulfide, distinct from structural and catalytic roles. This work establishes the vulnerability of human coronaviruses to two new thiol-based reducing agents, laying the groundwork to advance the field of thiol-based chemical probes for SARS-CoV-2 in the realms of target selection and site-specific redox assessment.

## Results

### Thiol-Based Reducing Agents Have Antiviral Activity against Human Coronaviruses.

The antiviral activities of P2119 and P2165 were first evaluated in a recombinant infectious clone of SARS-CoV-2 virus produced in human nasal epithelial (HNE) cells (Fig. 1*C*) (26). Exposure of SARS-CoV-2 to monothiol P2119 or dithiol P2165 reducing agents at 30 mM [note: this concentration was chosen based on reducing activity and effective surface airway deposition in mouse lungs (22)] resulted in respective >3 log and >6 log decreases in viral titer. To contextualize these findings, we compared dose-dependent virucidal activities of NAC, the gold-standard for approved care in thiol-based reducing agents, with P2165 against SARS-CoV-2 (Fig. 1*D*). Dose-dependent inhibition by P2165 was noted at concentrations over 3 mM (1 to 3 logs), while NAC was ineffective as a virucidal agent at concentrations up to 100 mM.

Analogous virucidal activities of thiol reducing agents were observed for the closely related coronavirus SARS-CoV (Fig. 1*E*), which shares ∼75% identity with SARS-CoV-2 spike protein and all four conserved disulfides in the RBD (*SI Appendix*, Fig. S1) (27). Coronavirus NL63-CoV (28) also utilizes ACE2 as its receptor (29) and has RBD disulfides that are essential for protein stability and receptor binding (*SI Appendix*, Fig. S1) (30). Exposure of NL63-CoV to P2119 (10 or 30 mM) or P2165 (10 mM) titered on LLC-MK2 cells produced similar decreases of viral titer (>3 to 4 logs) (*SI Appendix*, Fig. S2*A*). Finally, since glycosylation of the spike protein likely varies with cell type (31), the activity of thiol-based reducing agents was also tested with SARS-CoV-2 propagated in Vero E6 cells (*SI Appendix*, Fig. S2*B*). Here, P2119 decreased SARS-CoV-2 titers by ∼1 and 3 logs at 10 and 30 mM, respectively. P2165 decreased virus titers by ∼2 and 4 logs at 10 and 30 mM, respectively. These collective data indicate that P2119 and P2165 possess antiviral activity irrespective of the cell type producing SARS-CoV-2, that the dithiol is a more effective virucidal agent compared to the monothiol, and hint at a common mechanism of action for these thiol-based reducing agents.

### Thiol-Based Reducing Agents Inhibit SARS-CoV-2 Spike Binding to Human ACE2 Receptor.

**Fig. 2.**
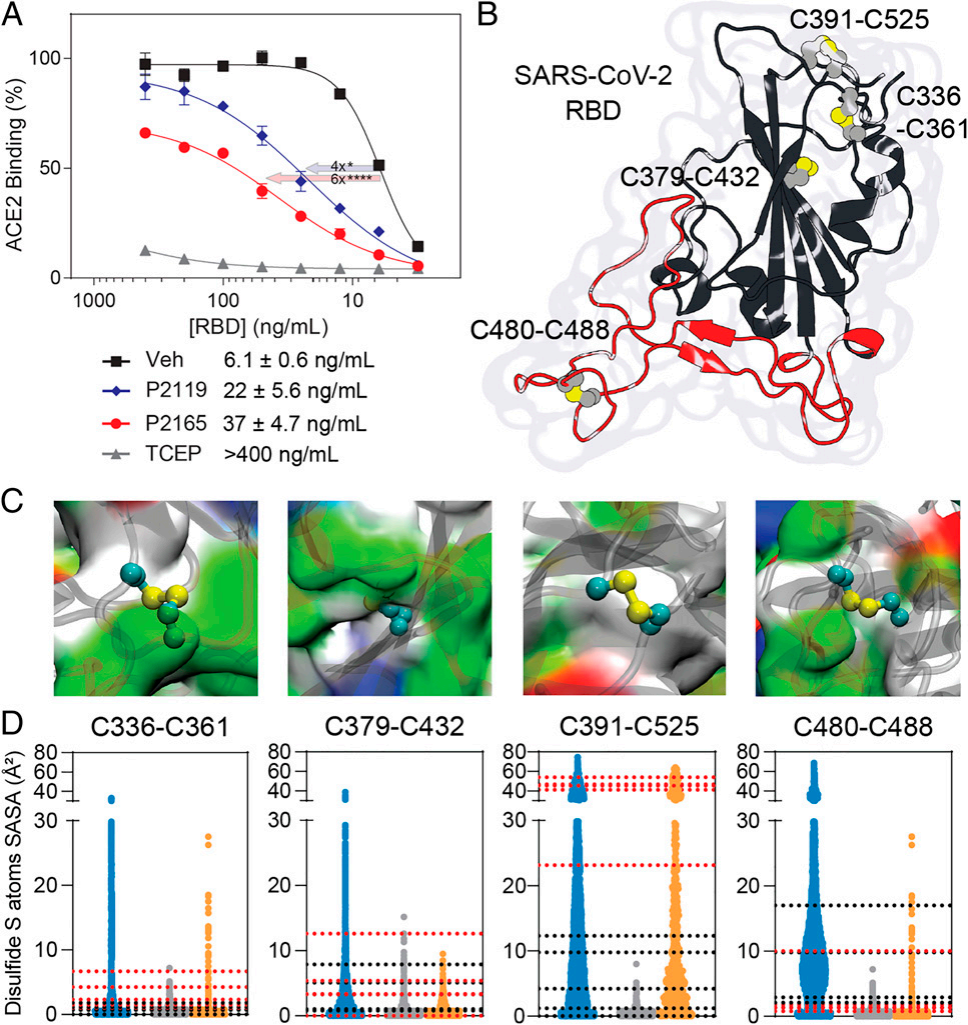
Disulfides of SARS-CoV-2 spike RBD. (*A*) Binding of recombinant SARS-CoV-2 spike RBD to immobilized ACE2. Quadruplicated experiments were performed at 4 °C in air-equilibrated solutions with RBD pretreated by PBS vehicle (square), 10 mM P2119 (diamond), P2165 (circle), or TCEP (triangle). Fitted EC_50_ values and SEs were listed and analyzed by ANOVA with Dunnett’s test. *P < 0.05; ****P < 0.0001. (*B*) Crystal structure of SARS-CoV-2 RBD showing four disulfides and RBM, colored in red. (*C*) Protein surface view illustrating the microenvironment of each RBD disulfide and colored by residue type: white, hydrophobic; green, polar; red, acidic; blue, basic. (*D*) SASA (Å^2^) calculated for RBD disulfides. Blue distributions correspond to isolated RBD MD simulations, gray to averaged three monomers in closed conformation and orange to open conformation from MD simulations performed for the entire spike glycoprotein trimer. Dotted lines correspond to calculated SASA values from different cryoelectron microscopy spike glycoprotein structures (black, closed conformations, PDB ID codes 6XM5, 7KRS, 7BNM; red, open conformations PDB ID codes 6XM3, 6XM4, 7KRR, 7BNN, 7BNO).

P2119 and P2165 are *p*-methoxybenzyl thiols conjugated to glucose (P2119) or mannose (P2165) monomers. The sugar units impart hydrophilicity and block diffusion of reducing agents into the cell where viral enzymes (e.g., proteases and mRNA polymerase) hijack host machinery. Since P2119 and P2165 are membrane impermeant and decreased titers were observed when the virus was exposed to these compounds prior to cell infection, we hypothesized that P2119 and P2165 acted by inhibiting viral entry. To examine this possibility, we assessed in vitro binding between recombinant spike RBD from SARS-CoV-2 and immobilized human ACE2. In this workflow, spike RBD was exposed to thiol-based reducing agents, subjected to spin gel filtration for small-molecule removal, and tested for ACE2 receptor binding. Compared to vehicle, P2165 and P2119 treatment shifted the half maximal effective concentration (EC_50_) for ACE2-RBD binding by roughly sixfold and fourfold, respectively, while the powerful phosphine-based reducing agent, Tris(2-carboxyethyl)phosphine (TCEP) completely blocked the binding of RBD to ACE2 (Fig. 2*A*). These data show that reduction of disulfides in spike RBD decreases binding to the ACE2 receptor and, by extension, is likely to hamper the ability of this virus to infect host cells. Interestingly, the trend in receptor-spike binding (i.e., TCEP ≫ P2165 > P2119) suggests that antiviral activity is correlated to the intrinsic reducing activity of reducing agents.

The globular-shaped SARS-CoV-2 RBD consists of several unstructured or poorly structured regions surrounding a β-sheet domain core and in its native state has four disulfide bonds (Fig. 2*B* and *SI Appendix*, Fig. S1). The microenvironment of each disulfide is very different in terms of amino acid sequence and physicochemical properties (Fig. 2*C*). RBD flexibility produces complex dynamic motions for all disulfides, as illustrated by the solvent accessible surface area (SASA) distributions obtained by MD simulations of either the isolated RBD domain or the entire spike glycoprotein ectodomain in three-down (closed) or one-up (open) RBD conformations (Fig. 2*D*) (32). In this analysis, the most buried disulfides were Cys336–Cys361 and Cys379–Cys432, while Cys391–Cys525 and Cys480–Cys488 were the most solvent accessible with isolated and open RBD structures showing comparable SASA values. Notably, similar microenvironment diversity is also captured by comparing spike structures solved under different experimental conditions.

### Mapping Redox-Sensitive Disulfides in Human and SARS-CoV-2 Cysteinomes in Native Virus.

**Fig. 3.**
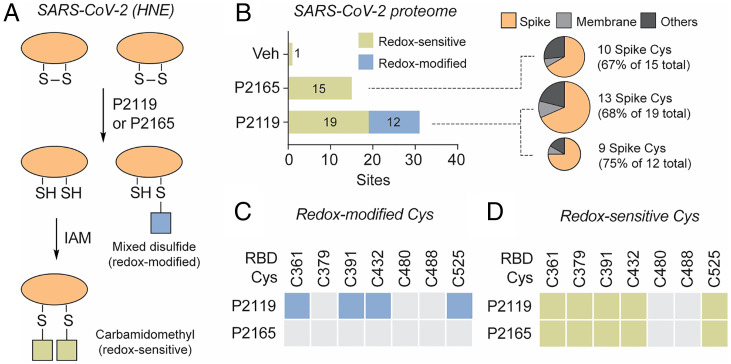
Cysteines in SARS-CoV-2 proteomes targeted by reducing agents. (*A*) Workflow for identifying redox-sensitive and redox-modified cysteines in SARS-CoV-2 (depicted in *SI Appendix*, Fig. S3*A*). HNE cultures were infected with SARS-CoV-2 D614G and diluted in PBS to an MOI of 0.1 on the apical surface. At 96 h postinfection, apical washes were incubated with PBS vehicle, P2119 (30 mM), or P2165 (30 mM). Samples were desalted, concentrated, alkylated by IAM, and processed using standard proteomic procedures. (*B*) Number of peptides with redox-sensitive or redox-modified cysteines in viral proteome. Protein compositions of reactive peptides are depicted in the pie charts. (*C* and *D*) Redox-modified and redox-sensitive RBD cysteines of native spike. Colored boxes indicate modified or redox-sensitive sites and gray boxes indicate unmodified/unidentified sites.

Based on the promising antiviral studies above, we next sought to qualitatively identify disulfides that exhibit sensitivity to thiol-based reducing agents in extracellular human and SARS-CoV-2 proteomes. Supernatants from cultured HNE cells infected with SARS-CoV-2 were exposed to P2119 or P2165, and subjected to reduction-alkylation redox proteomics (Fig. 3*A* and *SI Appendix*, Fig. S3*A*). Cysteine-containing peptides that undergo alkylation by iodoacetamide (IAM) after reduction with P2119 or P2165 represent targets of these thiol-based reducing agents (“redox-sensitive Cys”). In addition, monothiol compounds, like P2119, can form mixed disulfides with cysteines in protein targets (“redox-modified Cys”), whereas dithiols, like P2165, are designed to undergo rapid thiol-disulfide exchange to minimize stable covalent modification by the reducing agent.

Considering the SARS-CoV-2 proteome, only 1 cysteine-containing peptide was identified in vehicle-treated samples, whereas 15 and 19 redox-sensitive cysteine-containing peptides were identified in P2165- and P2119-treated samples, respectively (Fig. 3*B* and Dataset S1). In terms of redox-modified cysteines, 12 viral peptides were identified as covalently modified by P2119, whereas no stable covalent adducts with P2165 were identified. In addition, we identified redox-sensitive and -modified cysteines from the human proteome (*SI Appendix*, Fig. S4*A*), which originated from extracellular proteins and proteins from broken cells as artifacts. Of 46 cysteine-containing SARS-CoV-2 peptides, the majority were localized to spike protein: that is, 10 of 15 (67%) peptides after P2165 treatment, 13 of 19 (68%) peptides after P2119 treatment, and 9 of 12 (75%) P2119-modified peptides (Fig. 3*B*). RBD Cys361, Cys391, Cys432, and Cys525 were susceptible to covalent modification by P2119 (Fig. 3*C*), while most RBD disulfides were sensitive to both P2119 and P2165 (Fig. 3*D*). The peptide containing Cys480 and Cys488 was not identified unless the reduction was carried at an elevated temperature (*SI Appendix*, Fig. S4*B*), implying that accessibility may be a determinant of labeling in the context of the trimeric spike structure. These data indicate that thiol-based reducing agents preferentially target the spike protein relative to other exposed viral proteins, and that binding site disulfide Cys480–Cys488 is less susceptible to reduction in a native environment.

### Mapping Redox-Sensitive Disulfides and Reactive Cysteines in Recombinant SARS-CoV-2 RBD.

**Fig. 4.**
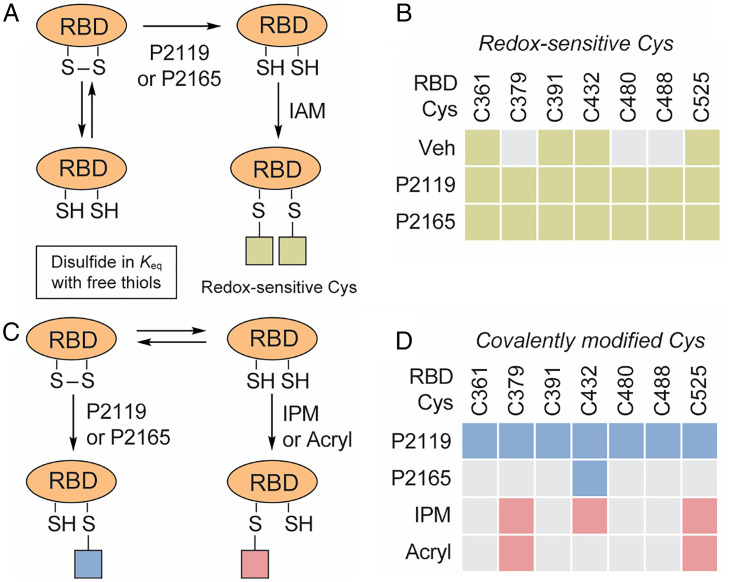
Redox-sensitive, redox-modified, and hyperreactive cysteines in SARS-CoV-2 RBD. (*A*) Workflow for identifying redox-sensitive cysteines in recombinant RBD of SARS-CoV-2 spike. (*B*) Redox-sensitive cysteines in recombinant RBD. Redox-sensitive and unmodified/unidentified sites are denoted by colored and gray boxes, respectively. (*C*) Workflow for identifying redox-modified and hyperreactive cysteines in in recombinant RBD. (*D*) Redox-modified and hyperreactive cysteines in recombinant RBD. Redox-modified, hyperreactive, and unmodified/unidentified sites are denoted by blue, pink, and gray boxes, respectively (workflow depicted in *SI Appendix*, Fig. S3 *C* and *D*).

The goal of subsequent proteomics was to identify “redox-sensitive” disulfides and “hyperreactive” cysteines in recombinant SARS-CoV-2 RBD (Fig. 4 *A* and *B*) and cross-reference these data to those obtained with intact SARS-CoV-2. Mass spectrometry (MS) analysis of vehicle-treated RBD identified peptides containing Cys361, Cys391, Cys432, and Cys525. In contrast, exposure to P2165 or P2119 identified peptides containing seven of eight RBD cysteines (Fig. 4*B*) (i.e., only the Cys336 peptide was not found). These mapping data and existing structures therefore suggest that Cys480–Cys488 forms a stable disulfide in recombinant RBD expressed and purified from human HEK293 cells. Notably, this disulfide pair is susceptible to reduction in truncated protein, in contrast to full-length spike harvested from infected cells. The remaining three disulfides Cys336–Cys361, Cys379–Cys432, Cys391–Cys525, exist as an ensemble of free and disulfide-bonded states.

To gain further insight into cysteines reactivity in recombinant RBD, we applied a reactivity-based approach using two different chemical probes for alkylation (Fig. 4*C*). In these studies, RBD was first alkylated with a very low concentration of iodo-*N*-(prop-2-yn-1-yl)acetamide (IPM; 0.1 mM) to detect low p*K*_a_ or “hyperreactive” cysteines (33). A modest (1 mM) level of dithiothreitol (DTT) was employed to reduce reversibly oxidized cysteines. After reduction, nascent thiols were alkylated with a high concentration of IAM (4 mM). MS analysis of peptides derived from these samples revealed that only Cys379, Cys432, and Cys525 were initially alkylated with IPM. Peptides containing these cysteines also exhibited more intense signals after DTT reduction-IAM alkylation, consistent with powerful thiolate nucleophilicity at these three sites (Fig. 4*D* and *SI Appendix*, Fig. S4*C*). In contrast, Cys361, Cys391, Cys480, and Cys488 were only alkylated after reduction with DTT. The reactivities of Cys379, Cys432, and Cys525 were further differentiated using an acrylamide electrophile (Acryl) derivatized from P2119, which is less reactive than IAM and IPM (*SI Appendix*, Fig. S3*D*). This compound underwent Michael addition with Cys379 and Cys525, but not Cys432 (Fig. 4*D*), suggesting that inherent nucleophilicity distinguishes these two cysteines in the RBD. In comparison, all RBD cysteines except for Cys336 were covalently modified by thiol nucleophile P2119, whereas only Cys432 formed covalent linkage with P2165 (Fig. 4*D*). In summary, findings from cysteine reactivity mapping of recombinant SARS-CoV-2 RBD are fully consistent with those obtained from reducing agent-treated samples, as well as peptides identified in viral infection studies. Importantly, Cys432, Cys379, and Cys525 may be distinct from other cysteines in the RBD from the standpoint of reactivity and accessibility. In particular, acrylamide-reactive Cys379 and Cys525 could be the nucleophilic “attacking” thiols of the corresponding disulfides.

### Computational Analysis of Disulfide Dynamics in the SARS-CoV-2 RBD.

**Fig. 5.**
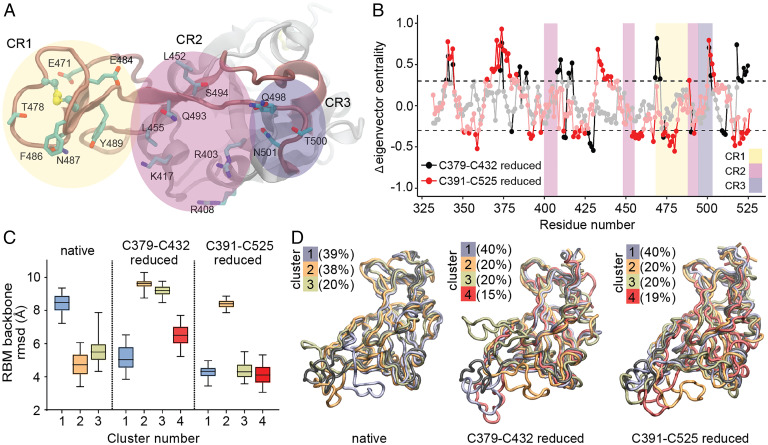
MD simulation of SARS-CoV-2 RBD disulfides. (*A*) Detailed representation of RBM residues in RBD that directly interact with ACE2, grouped in three contact regions (CRs). (*B*) Eigenvector centrality difference between reduced and native states. CR regions are colored as in *A*. Residues two σ-units away from normal distribution (dashed line) are shown. (*C*) Distribution of the root mean square deviation (rmsd) with respect to the experimental RBM structure for each ensemble of clustered structures. (*D*) Superposition of the reference RBD structure (black) with a representative RBD structure of each cluster ensemble from different simulations. The percentage of structures belonging to each cluster is indicated.

Among mobile regions in the RBD is the RBM that includes all RBD amino acids directly participating in the RBD–ACE2 interaction (Fig. 2*B*). The RBM is critical, as the plasticity of this motif is related to the ability to recognize and bind ACE2 (34). Prior studies have characterized residues directly involved in RBD–ACE2 interactions (8, 35, 36) and these residues have been parsed into three distinct “contact regions” (CR1–3) (Fig. 5*A*) (37). To dissect the relative contributions of experimentally observed disulfides to dynamics in CR1–3, we conducted MD simulation of the RBD in different redox states. The difference in eigenvector centrality (37) between native and reduced systems on a per residue basis is depicted in Fig. 5*B*. This analysis facilitates the identification of residues and regions that present significant changes from the mean dynamic behavior in the native-state postdisulfide reduction. The resulting data indicate that reduction of the Cys379–Cys432 disulfide bond leads to important dynamic changes in several residues of the interaction region, particularly in CR1. Reduction of the Cys391–Cys525 disulfide was associated with fewer changes overall, but some residues at CR2 and CR3 were also perturbed.

These data were supported by clustering trajectories using a structure similarity criterion (Fig. 5 *C* and *D*). The RBM region in native RBD was able to access conformations that differed significantly from a reference structure of this domain in complex with ACE2. Reduction of the Cys379–Cys432 disulfide resulted in structure ensembles that differed greatly from the reference structure. Even though reduction of Cys391–Cys525 generated structure ensembles comparable to native RBD in terms of a gross structural descriptor, the observed conformations were quite different. A greater tendency to “shrink” the RBM region approaching CR1 and CR3 was observed, which may have a significant impact on ACE2 recognition; a similar trend was observed for Cys480–Cys488. Although this disulfide pair is located at the RBM, its reduction yielded structure ensembles resembling not only native RBD, but also a subset exploring quite different RBM conformations (*SI Appendix*, Fig. S5).

In summary, MD simulations suggest that both Cys379–Cys432 and Cys391–Cys525 disulfides, experimentally confirmed by MS as targeted by reducing agents P2119 and P2165, play a major role in RBD dynamics. From a mechanistic standpoint, reduction of Cys379–Cys432 and Cys391–Cys525 disulfide pairs appears to impact RBD binding to ACE2, despite being structurally distant from the interaction region.

### Docking Thiol-Based Reducing Agents with the RBD of SARS-CoV-2.

**Fig. 6.**
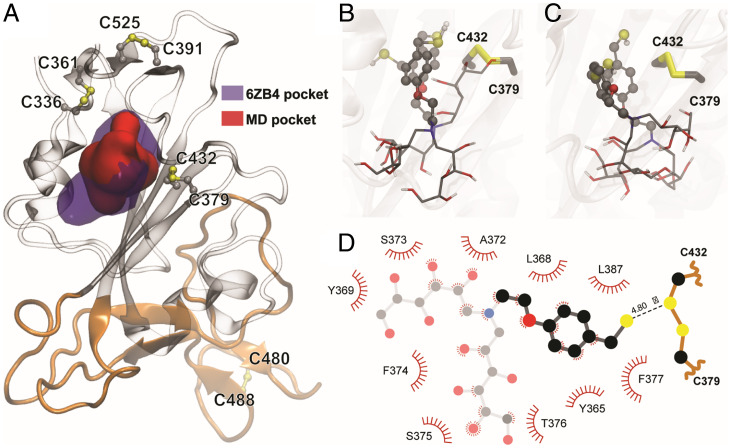
Reducing agents dock in a conserved hydrophobic pocket in the vicinity of C379–C432. (*A*) Representation of the hydrophobic pocket in the vicinity of the Cys379–Cys432 disulfide bond. Violet surface corresponds to the pocket identified in the presence of linoleic acid (PDB ID code 6ZB4) and the red surface shows a representative pocket identified during the RBD MD simulations. The RBM is colored orange. (*B* and *C*) Superimposed poses of P2119 and P2165 (transparent) docking, selected between the best ranked conformations, using as receptor the RBD of PDB ID code 6ZB4 structure in *B*, or representative conformation from the MD simulation in *C*. (*D*) Detailed two-dimensional map highlighting P2119-RBD contacts.

A hydrophobic binding pocket granting access to the Cys379–Cys432 disulfide was identified in different spike structures at the interface of neighboring RBD subunits. This pocket, shown in Fig. 6*A*, associates with hydrophobic molecules (e.g., linoleic acid), even in the closed state of SARS-CoV-2 and related coronavirus spike proteins (38–40). The binding of hydrophobic molecules to this pocket has been related to stabilization of spike protein in its closed conformation (38, 41), and ligands such as poly-unsaturated fatty acids or lipid-soluble vitamins have shown to inhibit SARS-CoV-2 RBD-ACE2 binding in vitro (42). When analyzing the dynamic behavior of the isolated RBD or its open conformation in the spike protein after extensive MD simulations, this pocket proved to be cryptic, characterized by breathing-like dynamics that allow transient accessibility to the Cys379–Cys432 disulfide (*SI Appendix*, Fig. S6). Available spike structures with hydrophobic ligands bound in this region indicate that larger pocket openings become accessible after binding. Indeed, cosolvent MD simulations from ligand-free systems showed that aromatic moieties, such as the benzyl group in P2119 and P2165, can bind via induced fit into this intriguing pocket regardless of the glycosylation or conformational state of the RBD (43).

To explore potential binding modes in greater detail, we evaluated different pocket conformations for docking experiments, including a well-defined pocket due to the presence of a ligand (linoleic acid, Protein Data Bank [PDB] ID code 6ZB4) and conformations from our MD simulations (Fig. 6*A* and *SI Appendix*, Table S1). Using isolated RBD as the receptor template for docking experiments representative of spike open conformations, we found that among the best ranked complex poses were those that allocated P2119 and P2165 to the previously mentioned pocket. In these poses, the benzene moiety of the P2119/P2165 compounds is buried in the protein core, and the thiol points directly toward Cys432 with distances between reducing agents and Cys432 sulfur atoms being less than 5 Å (Fig. 6 *B* and *C*). Several plausible interactions were observed in the docked reducing agent–spike complex. Specifically, the *p*-methoxybenzyl unit forms aromatic interactions with Phe377 and hydrophobic interactions with Leu368/Leu387. The hydrophilic polyol binding is stabilized by polar interactions and hydrogen bonds to RBD backbone carboxyl and amide groups outside the hydrophobic pocket (Fig. 6*D* and *SI Appendix*, Fig. S7). Docking poses obtained for both reducing agents were comparable, with only minor differences in the hydroxylated carbon tail conformations. Utilizing RBD structures derived from the native-state MD simulations, in which the pocket has a smaller cavity volume, yielded similar results (Fig. 6). Small differences in benzyl orientations were observed, but close distances were consistently maintained between corresponding sulfur atoms (*SI Appendix*, Table S1). It is worth noting that binding of these reducing agents, as well as other hydrophobic ligands, may rely on the spike open/partially open conformational state, as there are no large channels or cavities connecting this pocket with the protein surface in the closed state of the spike glycoprotein.

## Discussion

Although several thiol-containing compounds have been shown to inhibit viral receptor binding in vitro (44, 45), they lack potency (e.g., NAC or glutathione [GSH]), or are cytotoxic (e.g., DTT or TCEP). In this study, we characterized more recently synthesized P2119 and P2165 compounds as prototypic thiol-reductants for activity against human coronaviruses, including SARS-CoV-2, which exhibited activities comparable to or greater than reported for neutralizing antibodies (NAbs) (46, 47). Despite being administered at millimolar concentrations to maintain a necessary redox potential, these compounds have established safety profiles through aerosol administration in animals at doses that bracket those used in this study (22, 48), confirming that these concentrations can be pharmacologically relevant. Their virucidal effects allowed comparisons of the intrinsic reducing ability of the thiol group and the ability to form stable covalent linkages. Furthermore, their ability to impair ACE2 binding correlates to the virucidal effect, consistent with what has been shown in NAbs (49). Based on their restricted location of action (i.e., the extracellular compartment), the ability of P2119 and P2165 to inhibit RBD-ACE2 binding in vitro, and qualitative proteomic cysteine site-reactivity mapping in SARS-CoV-2, the simplest explanation for the observed antiviral activity of these agents is that they efficiently reduce key disulfides in SARS-CoV-2 required for infectivity. However, there is certainly “off-target” reduction of host proteins in parallel. Whether these off-target effects are tolerable to the host must be assessed from the therapeutic index of these compounds, which will be formally quantitated in future pharmacology and inhaled toxicity studies.

Only four proteins (Dataset S1) from the entire SARS-CoV-2 proteome were reactive to reductive treatments. These proteins included the spike (S) and membrane (M) proteins, which are the most protruding or abundant proteins in the virion envelope (E), respectively (the molar ratio of E:S:M in the lipid bilayer is ∼1:20:300) (50); and two accessory proteins ORF8 and ORF7a, which are expressed by infected cells as transmembrane or secreted proteins, respectively (51). The observation that most mapped cysteine modifications (>65%) occurred on the spike protein supports our proposed mechanism of action for P2119 and P2165 to block infectivity. Of particular importance is the reduction of two RBD disulfides, Cys379–Cys432 and Cys391–Cys525, which were identified as redox-sensitive in both native spike protein and recombinant RBD. Despite the susceptibility of the Cys480–Cys488 pair to reduction in recombinant RBD, this disulfide was not in-play in native virus during infection. In essence, the Cys480–Cys488 disulfide is a conserved, thermodynamically stable disulfide, while Cys379–Cys432 and Cys391–Cys525 disulfides are in dynamic equilibrium with their thiol states and, thus, are more sensitive to changes in redox poise.

MD simulations carried out in this study indicate significant conformational changes in the RBM concomitant with reduction of Cys379–Cys432 and Cys391–Cys525 disulfides, even though they are distal to the ACE2 recognition motif. The dynamic effects seem to be more prominent regarding the Cys379–Cys432 pair, in agreement with deep mutational scanning information (36). These RBD disulfides appear to be allosteric, a class of disulfides distinct from structural or catalytic disulfides that control protein function by triggering conformational change when they break or form (52). The impact of this effect at other regions in the spike remains to be elucidated. Experimental data throughout this study also distinguished Cys379–Cys432 and Cys391–Cys525 disulfides, including observed redox-dependent changes in cysteine reactivity and mixed disulfide formation with P2119. Furthermore, ligand docking situated P2119/P2165 compounds in similar hydrophobic pockets with access to the Cys379–Cys432 disulfide. Predicted binding poses were supported by the unexpected covalent linkage between Cys432 and P2165 (Fig. 4*D*), likely caused by restricted movement and limited solvent access of the resolving thiol in the binding pocket, such that base-promoted ring closure did not occur. Although covalent modification of viral cysteines is not required for the antiviral potency of reducing agents, our docking studies outline ostensible interactions between P2119/P2165 and RBD, which could be exploited in future works for evaluating compounds’ affinities with RBD and rationally modifying them to enhance RBD targeting.

Bjorkman and coworkers (53) conducted a structural comparison of SARS-CoV-2 spike NAbs and showed NAbs either directly bind to the RBM or their epitopes, including part of the RBM. For example, a group of NAbs primarily interact with Arg346 and Asn440, which are subject to NAb escape when mutations occur. A report by Wilson and coworkers (54) showed that binding and neutralization of the two most common antibody families were abrogated by Lys417Asn and Glu484Lys mutations. Disruption of disulfide bonds adjacent to the aforementioned NAb binding sites (e.g., Cys336–Cys361 and Cys379–Cys432) could be an alternative mechanism to block ACE2 binding with spike RBD, as evidenced by the potency of P2119 and P2165 against the SARS-CoV-2 Asp614Gly mutant distinguished by an increased level of functional spike, enhanced infectivity, and reinforced ACE2 binding (55–57). No SARS-CoV-2 variant of interest or of concern reported so far, presents mutations of cysteine residues forming the disulfide bonds studied herein, underscoring the conservation of cysteine residues in the SARS-CoV-2 spike structure, dynamics, and function. However, mutations structurally close to disulfides are present in different variants of concern, such as the Glu484Lys and Asn501Tyr found in B.1.1.7 (alpha), B.1.351 (beta), and P.1 (gamma), the Glu484Gln found in B.1.617 (delta), or the Ser375Phe, Thr478Lys, and Glu484Ala present in B.1.1.529 (omicron). As variants emerge and pose new threats, targeting spike disulfides offers a variant-independent approach for the control of the ongoing and future COVID-19 pandemics.

In summary, thiol-based reducing agents may derive antiviral activity in two ways: 1) direct reduction of disulfides on redox-sensitive target proteins required for viral entry (e.g., RBD Cys379–Cys432 and Cys391–Cys525), and 2) modulating the extracellular redox poise required for SARS-CoV-2 entry into cells (58). In addition, in vivo P2119/P2165 may also reduce oxidized components of the host airways epithelial lining fluid (59); for example, the increased amounts of GSH disulfide (GSSG) generated during inflammation (60, 61) associated with a SARS-CoV-2 infection, and help minimize tissue oxidative damage. The data generated from the two complementary glycosylated thiols (P2119, P2165) have identified key cysteine pairs in SARS-CoV-2 spike protein that, when reduced, block SARS-CoV-2 infection and permitted analyses of the requirements for reducing agent intrinsic activity and covalent linkage. These data provide a roadmap to develop thiol-based reagents with the necessary pharmacokinetic profiles and therapeutic indices targeting vulnerable disulfide bonds in SARS-CoV-2.

## Methods

Methods and additional data and figures, including virus propagation and inhibition assay, RBD-ACE2 binding assay, LC-MS/MS sample preparation and analysis, docking, and MD simulations, are provided in *SI Appendix*.

## Supplementary Material

Supplementary File

Supplementary File

## Data Availability

All study data are included in the article and supporting information.
